# MSMHSA-DeepLab V3+: An Effective Multi-Scale, Multi-Head Self-Attention Network for Dual-Modality Cardiac Medical Image Segmentation

**DOI:** 10.3390/jimaging10060135

**Published:** 2024-06-03

**Authors:** Bo Chen, Yongbo Li, Jiacheng Liu, Fei Yang, Lei Zhang

**Affiliations:** 1 School of Mechanical, Electrical and Information Engineering, Shandong University, Weihai 264209, China; 202137544@mail.sdu.edu.cn (B.C.); 202017460@mail.sdu.edu.cn (Y.L.); 202200800027@mail.sdu.edu.cn (J.L.); 2 Department of Diagnostic Radiology and Nuclear Medicine, University of Maryland School of Medicine, Baltimore, MD 21201, USA; cszhanglei@gmail.com

**Keywords:** cardiac segmentation, medical image, multi-head self-attention mechanism, multi-scale, dual model

## Abstract

The automatic segmentation of cardiac computed tomography (CT) and magnetic resonance imaging (MRI) plays a pivotal role in the prevention and treatment of cardiovascular diseases. In this study, we propose an efficient network based on the multi-scale, multi-head self-attention (MSMHSA) mechanism. The incorporation of this mechanism enables us to achieve larger receptive fields, facilitating the accurate segmentation of whole heart structures in both CT and MRI images. Within this network, features extracted from the shallow feature extraction network undergo a MHSA mechanism that closely aligns with human vision, resulting in the extraction of contextual semantic information more comprehensively and accurately. To improve the precision of cardiac substructure segmentation across varying sizes, our proposed method introduces three MHSA networks at distinct scales. This approach allows for fine-tuning the accuracy of micro-object segmentation by adapting the size of the segmented images. The efficacy of our method is rigorously validated on the Multi-Modality Whole Heart Segmentation (MM-WHS) Challenge 2017 dataset, demonstrating competitive results and the accurate segmentation of seven cardiac substructures in both cardiac CT and MRI images. Through comparative experiments with advanced transformer-based models, our study provides compelling evidence that despite the remarkable achievements of transformer-based models, the fusion of CNN models and self-attention remains a simple yet highly effective approach for dual-modality whole heart segmentation.

## 1. Introduction

According to statistics from the World Health Organization, mortality from cardiovascular diseases accounted for 25% of that in developing countries and 50% of that in developed countries in the early 21st century. Cardiovascular diseases result in 19 million deaths every year all over the world [1]. Cardiovascular diseases have become increasingly obvious as lifestyles change, population aging accelerates, and cardiovascular diseases occur at a younger age. Medical imaging serves a notable role in medical diagnosis, and the medical imaging methods that are most widely applied now include ultrasound, computed tomography (CT) [2], and magnetic resonance imaging (MRI) [3]. Locating lesions of the heart using noninvasive CT and MRI acts as a vital examination approach for the clinical diagnosis of cardiac diseases. Accurately distinguishing the structures of different substructures of the heart is the basis for the quantitative analysis of global and regional cardiac function and the detection and diagnosis of cardiac diseases [4]. The conventional method of manually analyzing the images comes with severe limitations; the process is time consuming, tedious, and greatly affected by how experienced the analyst is. For this reason, discovering an accurate, rapid, and automatic heart segmentation method has become a research hotspot in this field. Medical image segmentation refers to the process of segmenting a medical image into several mutually disjoint “connected” regions based on a certain similarity feature of the medical image (such as the brightness, color, texture, area, shape, location, local statistical characteristics, or spectral characteristics). This technique has extensive application and research value in fields such as clinical diagnosis, pathological analysis, surgical planning, image information processing, and computer-assisted surgery. The relevant features are consistent or similar within a region but clearly vary in different regions. In other words, pixels on the region boundaries are quite discontinuous. Medical image segmentation is the basis for various medical imaging applications, and relevant technologies have manifested, increasing the clinical value in current clinical auxiliary diagnosis, image-guided surgery, and radiation therapy [5]. Semantic segmentation is defined as the process of segmenting a scene image into several meaningful image regions and assigning designated labels to different image regions. Traditionally, the semantic segmentation of images is achieved by segmenting images into different regions based on the boundary, color, texture, and other features in the images, with classical threshold-, boundary-, clustering-, and graph theory-based segmentation commonly applied. Image segmentation technologies are only applicable for the processing of grayscale images due to the limitations of computer hardware in the early stage of the technology’s development. With gradual development over time, image segmentation technologies can now be applied to the processing of RGB images.

Regarding existing image segmentation networks, the mode of encoding by down-sampling and decoding by up-sampling is usually used, and the simultaneous jumping connection of up-sampling results with shallow down-sampling features enables the maximum preservation of original image features. In this way, however, only simple pixel classification is achieved, and global information is not considered. Good image segmentation results can be obtained through the combination of atrous convolution and multi-scale information, but the information extracted by such methods is only limited to a specific range, and global information of a wider range cannot be acquired. Self-attention mechanisms applied in the early days of the field of natural language processing are proposed in the transformer model, in which many attention mechanisms (AMs) are used as the encoder–decoder framework. With self-attention mechanisms, global information can be effectively used, and the relationship vector of an input with all other inputs can be concurrently calculated. The efficiency of feature extraction using a 5-layer self-attention mechanism is approximately equal to that of using 30 convolutional layers.

To acquire larger global visual fields, a self-attention mechanism module was introduced into the DeepLab V3+ network in this study to acquire global feature relationships, and the accuracy of segmentation of different objects was adjusted by changing the size of images segmented. In addition, the effects of self-attention mechanisms at different scales on segmentation results were fully investigated, and a whole heart segmentation solution intended to segment the whole heart into seven different substructures was put forward. Self-attention mechanisms were applied, respectively, for the different dimension features extracted during down-sampling using DeepLab V3+. It was revealed in the experiments that the network model proposed in this study improved the overall accuracy of the semantic segmentation results while further improving the accuracy of tiny structure segmentation by changing the self-attention scale; the convergence rate of the network was also greatly improved compared with that of the original network. The major contributions of this study are as follows:

1. Three MHSA mechanisms at different scales were designed and separately incorporated into the DeepLab V3+ network to perform an improved segmentation of features with different sizes, and thus, cardiac substructures are segmented more accurately.

2. The network structure proposed in this study also enhances the decoder by adding skip connections suitable for medical image segmentation to better recover the image boundary information.

## 2. Related Works

### 2.1. Supervised Image Segmentation

Targeting the shortcomings of convolutional neural networks (CNNs) in segmentation tasks, Long et al. proposed a fully convolutional network (FCN) for image segmentation [6]. The FCN is built on the AlexNet, in which all connected layers are converted into convolutional layers and the dimensions of feature maps are increased by up-sampling. However, the FCN has shortcomings, such as loss of image detail information and insufficient segmentation accuracy, which needs further improvement. Thereafter, a U-net architecture was proposed by Ronneberger et al. based on the concept of FCN [7]. The U-net structure comprises an encoding stage and a decoding stage. Part of the feature information of images is lost due to the continuous convolution kernel and pooling operation at the encoding stage, whereas detail features of images are enriched due to the fusion of feature maps after up-sampling at the decoding stage with the front-end information of the jumping connection. The U-net structure has been applied in medical image segmentation tasks for neurons, tumors, and HeLa cells. Afterwards, a modified U-net, called V-net, was specifically designed by Milletari et al. for 3D medical image segmentation [8]. Due to the 3D convolution technique and confidence segmentation index adopted, V-net is more suitable for medical image segmentation. In 2018, the Unet++ structure was proposed by Zhou et al. through innovation of the U-net structure [9]. Compared with the original U-net, Unet++ connects the first four layers of the U-net structure together and integrates them by feature superposition, and the network itself can learn the feature weights at different depths. In 2015, DeepLab V1 [10] was put forward by Chen et al., which, for the first time, uses VGG16-based DCNN with atrous convolution to increase receptive fields and obtain richer spatial semantic information, with an accuracy of 71.6% in the VOC 2012 dataset. DeepLab V2, proposed in 2017 [11], uses atrous spatial pyramid pooling (ASPP) to acquire global semantic information, realizing the extraction of features at different scales, greatly increasing the receptive fields and having an accuracy of 79.7% in the VOC 2012 test set. DeepLab V3 [12] and DeepLab V3+ [13] were successively put forward in 2017–2018. With DeepLab V3, the ASPP module was modified, the original 3 × 3 convolution module for rate = 24 was replaced with a 1×1 convolution module, and a mean intersection over union (MIoU) of 86.9% was achieved on the test set. And for DeepLab V3+, a decoder module was added to DeepLab V3, and the 4-fold down-sampling results obtained using the shallow feature extraction network were combined with the 4-fold up-sampling results of deep features subjected to 16-fold down-sampling and then extracted using the ASPP+ module, followed by 4-fold up-sampling to reduce to images to the same size as the original map, improving the operation rate and robustness of the network. The addition of the encoder also greatly improved the accuracy of the network in segmentation, with an MIoU of 89.0% in the VOC dataset. The DeepLab V3+ network also performed better in semantic segmentation using deep learning methods, but it still had disadvantages such as local information loss and inaccuracy in the segmentation of micro-objects.

### 2.2. Cardiac Medical Image Segmentation

In 2016, an FCN was used by Tran et al. for cardiac left ventricle (LV) and right ventricle (RV) segmentation tasks of MRI images for the first time [14], and it was found that the FCN is superior to the traditional automatic algorithm in multiple evaluation indexes in a series of heart datasets, pointing the way for the future development of whole heart segmentation. In 2017, Yang et al. tightly coupled an FCN with 3D operators, transfer learning, and depth supervision mechanisms to extract 3D context information [15]. This method performed excellently in simultaneously labeling the seven sub-structures of the heart in the Multi-Modality Whole Heart Segmentation (MM-WHS) Challenge 2017 dataset. In the same year, a network consisting of two FCNs was proposed by Payer et al. for multi-label whole heart segmentation of CT and MRI images [16]. The first FCN localizes the approximate center of the heart in lower resolution voxels, and the second one realizes final segmentation on higher resolution voxels based on the localization in the first stage. The encoding–decoding structure of U-net has been proven to be more suitable for the segmentation tasks of medical images. Xu et al., therefore, proposed the CFUN, the combining faster Region-CNN (R-CNN) and U-net network [17], in 2018. With the accurate localization ability of faster R-CNN and the strong segmentation ability of the U-net network, good segmentation results are obtained using the CFUN while significantly reducing the computational cost. Additionally, with the boundary information-based 3D edge-loss function as an auxiliary loss, the CFUN achieves an accelerated convergence rate of training, improved segmentation accuracy, and good segmentation performance.

### 2.3. Research and Application of AMs

Besides the convolution method for image feature extraction, some special AMs can also be applied to improve the efficiency of such extraction. AMs, which were first proposed by Treisman and Gelade in the 1990s [18], highlight or weaken the effects of inputs on outputs by assigning different weights to inputs, which are characterized by intuitiveness, generality, and interpretability and thus have now been widely applied in many fields of deep learning. In 2014, AMs were successfully applied in machine translation by Bahdanau et al. [19], achieving the accurate calibration of translation statements by decoding the last state of the multilayer perceptron to obtain the attention signal on the input sequence. In the same year, AMs were combined with recurrent neural networks (RNN) by the Google DeepMind team for image classification, with remarkable results achieved as well. Hence, AMs are continuously developing and being gradually applied in major fields. Visual AMs are generated since people learn to screen for the details that need to be focused on by observing things autonomously, searching for the details that are the focus of their attention and ignoring other useless information at the same time, all of which greatly enhance the accuracy and efficiency of visual information processing. AMs were first applied in visual tasks in 2015 by Xu et al. [20]. In 2017, an attention model incorporating a mask module was proposed by Wang et al. [21], forming a novel neural network framework with residual attention modules of mask and trunk branches. In 2018, two models based on channel attention and spatial attention were put forward by Roy et al. [22] to determine the most important regions in a channel and a space by calculating the weights of each region in the channel and space, respectively. In 2019, a dual attention network (DANet) [23] was proposed by the Institute of Automation, Chinese Academy of Sciences for natural scene image segmentation. In DANet, AMs are introduced in the spatial and channel dimensions of features, effectively grasping the global dependence of features. To further acquire the features of global dependence, the output results of the two modules are additively fused to obtain the final features for pixel classification.

A transformer model with self-attention as the basic unit was proposed by Vaswani et al. in 2017 [24], which is a successful application of a self-attention mechanism. As a kind of AM, self-attention mechanisms make the network model focus on information more important to current tasks, assigning weights according to the importance of information, highlighting the features beneficial to given tasks, and making the model concentrate more on local information, thereby enhancing the overall performance of the model. Swin-Unet for medical image segmentation put forward by Hu et al. in 2021 [25] is a pure transformer model-based U-net. In Swin-Unet, a hierarchical Swin-transformer with a moving window is used as the encoder to extract contextual features, and a symmetric Swin-transformer-based decoder with a patch expansion layer is employed for up-sampling to restore the spatial resolution of feature maps. Due to great interest in AMs in recent years, studies on AMs are also conducted constantly by scholars and researchers, so that they might be applied in more fields.

## 3. Materials and Methods

### 3.1. MHSA Mechanisms

#### 3.1.1. Self-Attention Mechanisms

Since the emergence of deep learning, deep CNNs have been the mainstream models in the computer vision field, with impressive achievements. CNNs have natural inductive biases for image issues, namely translation equivariance and locality. Since 2020, Transformer has made outstanding achievements in the field of computer vision, including image classification (ViT, DeiT), target detection (DETR, Deformable DETR), semantic segmentation (SETR, MedT), and image generation (GANsformer). Unlike convolution, self-attention, the core of Transformer, does not have fixed and limited receptive fields, but self-attention has the advantage of acquiring long-range information through attention. In contrast, CNNs must acquire larger receptive fields through the constant accumulation of convolutional layers.

**Figure 1:**
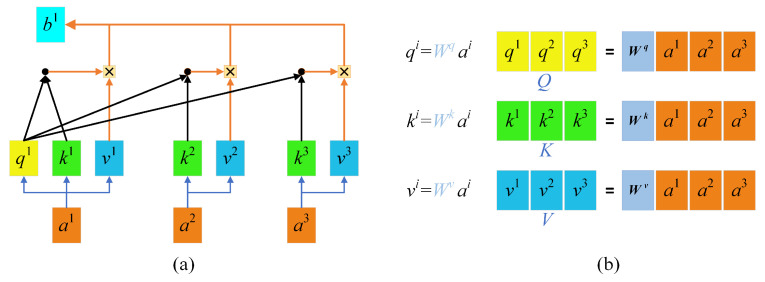
Self-attention mechanisms: (**a**) Self-attention mechanisms; (**b**) QKV weight matrices.

The principle of self-attention mechanisms is illustrated in Figure 1a. With self-attention mechanisms, three vectors, namely a query vector (*q*), a key vector (*k*), and a value vector (*v*), are first generated from the input vectors of each encoder. These three vectors are created by multiplying the input vector *a* by three weight matrices [Figure 1b]. The three vectors *q*, *k*, and *v* are then plugged into Formula (1) to obtain *b*^1^, the relationship vector of the input *a*^1^ with *a*^1^, *a*^2^, and *a*^3^. The self-attention mechanism can be represented by Formula (2), where $d_{k}$ is the number of columns of the *Q*, *K* matrix, namely, the vector dimension, which is divided by $\sqrt{d_{k}}$ to prevent an excessive internal product.

(1) $$b^{1}=\sum_{i}softmax(q^{1}k^{i})\cdot v^{i}$$

(2) $$Attention(Q,K,V)=softmax(\frac{QK^{T}}{\sqrt{d_{k}}})$$

In such a way, a relationship vector for each input against all inputs is acquired, which provides global information. This calculation method based on matrix operations improves the efficiency of computation. The calculated parameters can also serve as inputs and be input into the next self-attention computing module. According to experimental findings, the efficiency of feature extraction using a 5-layer self-attention mechanism is approximately equal to that of using 30 convolutional layers.

#### 3.1.2. MHSA Mechanism Framework

1. Main framework:

MHSA mechanisms are equivalent to the fact that different people have different views on the same thing, everyone thinks in their own way, and the nature of problems can be more easily understood as more people become concerned with those problems. An MHSA mechanism has many self-attention layers, and the input *X* is first transmitted to *h* different self-attention layers to obtain *Z*, a set of *h* output matrixes. Then, the *h* output matrixes in *Z* are stitched together (concat) using the MHSA mechanism and transmitted into a linear layer to acquire the final output, *Z*, of the MHSA. In this study, several $W_{k},W_{q},W_{v}$ variables were prepared to map original sentence vectors into different spaces, and the results in different spaces were then obtained by using the point multiplication operation with the query (*Q*), key (*K*), and value (*V*) [Formula (3)]. The number of spaces where vectors were mapped into was equal to the number of heads. The MHSA mechanism is shown in Formula (4).

(3) $$head_{i}=Attention(QW_{q},KW_{k},VW_{v})$$

(4) $$MultiHead(Q,K,V)=Concat(head_{1},...,head_{h})W$$

**Figure 2:**
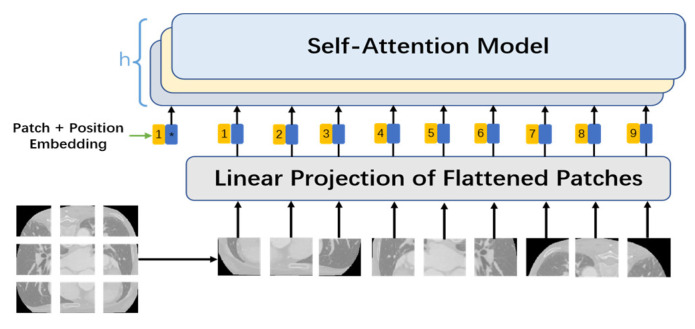
MHSA mechanism.

Unlike the self-attention mechanism in natural language processing, with a word as an input vector, the self-attention mechanism used in the network mode proposed in this study cut an original image into several smaller *n* × *n* image patches using convolution with a size of n × n and a step of n. These image patches were converted into patch embeddings, with upper position encoding added as the input part of the MHSA at the same time. The MHSA mechanism is displayed in Figure 2.

2. Patch embedding:

Patch embedding is analogous to sentence and word embedding of NLP. Because the Transformer’s inputs are a sequence of token embeddings, feature extraction can be performed after patch embeddings of the images are sent to Transformer. As for feature extraction using the self-attention mechanism, an original 2D image is first converted into a series of 1D patch embeddings, and the input 2D image is recorded as *x*, $x\in R^{H\times W\times C}$, where *H* and *W* are the height and width of the image, respectively, and *C* is the number of channels, which is 3 for RGB images. Next, the image is divided into patches with a size of n × n; that is, the image is cut into *N* = *HW*/*P*^2^ patches by convolution with a size of n × n and a step of n. Afterwards, the patches are flattened into features with a size of *D* = *P*^2^·*C*. After that, the patches are mapped to a D-sized dimension via a simple linear transformation to acquire the patch embeddings: $x_{p}\in R^{H\times W\times C}$.

3. Position embedding:

Retaining information about the relative position of each word in a sentence has been proven to be absolutely necessary in the field of natural language processing. Patches are processed sequentially by an RNN, so the position information of words in sentences is naturally retained. Unlike an RNN, self-attention mechanisms cannot retain position information, since every input vector is simultaneously input and concurrently computed. In cases where no position information from patches is available, the model must learn by puzzling through the patches’ semantics, which generates an additional learning cost, so the provision of position information makes learning relatively much easier. In the model proposed in this study, therefore, a special position embedding was required in addition to patch embedding for each patch. Position embedding added position information to the patch embedding vectors as well as a position vector on each patch. For the position embedding of each patch, the sine and cosine similarity of the position vector of the patch with other patches was calculated based on Formulas (5) and (6), where pos denotes the position vector of patches in the image, and *i* stands for the size of each patch.

(5) $$PE(pos,2i)=\sin(pos/1000^{2i/d_{model}})$$

(6) $$PE(pos,2i+1)=\cos(pos/1000^{2i/d_{model}})$$

4. Class token:

By referring to ViT, a class token with a dimension of class_nums × D was stitched into this study as a final image feature obtained by pixel segmentation, and a full convolutional layer was added to the final output feature to reduce it to a feature map that contained class_nums classifications and had the same size as the input image. The embedding corresponding to the class token was randomly initialized at training and then acquired through training.

### 3.2. MSMHSA-DeepLab V3+

**Figure 3:**
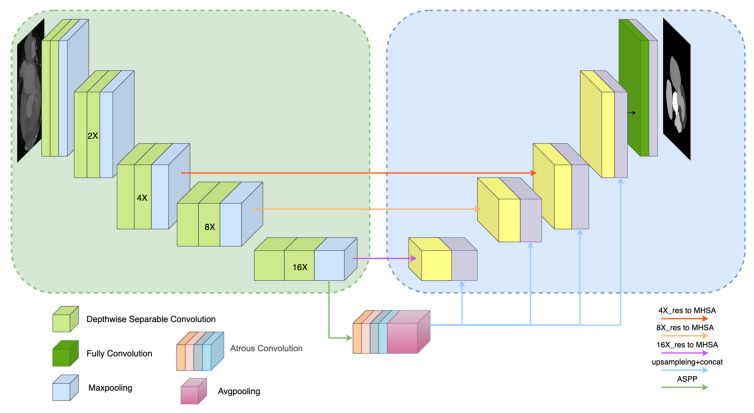
Overall structure of MSMHSA-DeepLab V3+ network.

According to the overall structure of the MSMHSA-DeepLab V3+ network proposed in this study, as shown in Figure 3, MSMHSA-DeepLab V3+ contained self-attention at different scales. The network model consisted of two modules, namely an encoder comprising the Xception network, the ASPP module, the MHSA mechanism module, and a decoder using jumping connection.

#### 3.2.1. Encoder

As shown in the left half of Figure 3, the encoder of the network structure contained a shallow feature extraction network and two deep feature extraction networks.

1. Xception:

In contrast with the shallow feature extraction network in DeepLab V3+ network, the Xception network, with a better effect and fewer parameters, was used as the shallow feature extraction network in the MSMHSA-DeepLab V3+ network. Unlike traditional convolution, which enabled the simultaneous extraction of channel features and spatial features, the depthwise separable convolution introduced into Xception achieves the separate extraction of channel and spatial features, solving the problem of excessive coupling of channel correlation and spatial correlation due to the traditional convolution operation. In addition, with fewer parameters and a smaller computational load, depthwise separable convolution also makes the network deeper in cases with the same number of parameters, thus obtaining a higher performance. Moreover, a residual error module introduced into Xception makes the feature information extracted by the model richer and avoids gradient descent caused by using an excessively deep network in the model training. In this study, Xception was employed for the first time to perform 16-fold down-sampling of input images and to send the down-sampling results into the ASPP module. The 16-fold, 8-fold, and 4-fold down-sampling results obtained during down-sampling were separately input into MHSA mechanisms at different scales. Finally, feature extraction networks for features of different sizes were acquired (Figure 3).

2. MSMHSA:

In medical images, there exists a fixed relative positional relationship between tissues, and certain tissue parts exhibit strong correlations. Due to the limitations of traditional feature extraction methods in the visual domain, this study introduces the MHSA (multi-head self-attention) mechanism, which can simultaneously extract global information to assist in semantic feature extraction from images.

In this study, a 12-layer self-attention mechanism module with 12 heads was utilized. The module underwent multiple down-sampling processes via Xception, each time with a down-sampling rate of 0.5×, resulting in 4×, 8×, and 16× down-sampled outputs. These outputs were then input into MHSA mechanisms with patch sizes of 4 × 4, 3 × 3, and 2 × 2, respectively, to obtain global semantic information feature maps at three different scales (corresponding to (a) 16×_res to MHSA network, (b) 8×_res to MHSA network, and (c) 4×_res to MHSA network in the figure).

**Figure 4:**
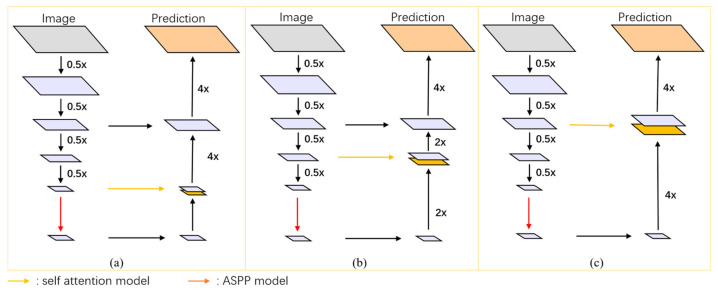
Refined structure of MHSA networks at different scales: (**a**) 16×_res to MHSA network, (**b**) 8×_res to MHSA network, and (**c**) 4×_res to MHSA network.

Subsequently, these three feature maps were concatenated with the output of the ASPP (Atrous Spatial Pyramid Pooling) module to acquire the final deep feature extraction images. By employing MHSA mechanisms with different patch sizes, three segmentation networks with similar structures but varying segmentation accuracies were obtained, as depicted in Figure 4.

3. ASPP

To ensure no excessive loss of detail features in global semantic feature acquisition using MHSA, the ASPP module was retained in this study. The feature maps extracted from the shallow feature extraction network were input into the ASPP module. In the ASPP module, the same feature is subjected to processing at different sampling rates through concurrently atrous convolution of different sampling rates, and final results are subjected to concat fusion to acquire the feature information at multiple scales of objects. The introduction of atrous convolution solves the contradiction between obtaining larger receptive fields and calculation quantities. With a convolution kernel of fixed size but different atrous rates, receptive fields of different sizes can be obtained with the calculation quantity unchanged, thereby acquiring object information at multiple scales.

In the Atrous Spatial Pyramid Pooling (ASPP) module, using uniformly spaced expansion coefficients offers several advantages. The primary benefit is its ability to more effectively capture multi-scale contextual information. By employing atrous convolutions with different dilation rates, the ASPP module can capture features at multiple scales without significantly increasing the number of parameters.

The choice of spacing for the expansion coefficients in the ASPP module should be based on the specific requirements of the task and the nature of the input data. An appropriate spacing size strikes a good balance between capturing fine details and broader contextual information. If the spacing is too small, the receptive field may not be large enough to capture the necessary context, resulting in a suboptimal performance in capturing larger structures. Conversely, if the spacing is too large, the model may miss critical details, which are essential for precise segmentation tasks.

In this study, the entire ASPP module is composed of atrous convolutions with expansion coefficients of 1, 6, 12, and 18, along with a global average pooling operation. This selection method is also widely adopted in other research works, such as DeepLabv3. By choosing these intermediate values, the model can effectively balance between capturing contextual information and fine details, providing a sufficiently large receptive field to capture rich contextual information while maintaining the ability to accurately capture finer details.

#### 3.2.2. Decoder

Considering the high effectiveness of encoding and corresponding decoding in medical image segmentation, in this study, U-net’s jumping connection and stepwise sampling were introduced in the decoder. The multi-scale fusion feature maps obtained using the ASPP module were subjected to concat fusion with the global semantic information feature maps acquired using the MHSA mechanism. Prior to concat fusion, the results obtained using the ASPP module were first transformed into feature maps of the same size as the results from the MHSA mechanism. After concat fusion, the obtained results were subjected to gradual up-sampling, during which the sampling results were subjected to jumping connection with the sampling results of the same dimension in the corresponding down-sampling in the encoder. Jumping connection is conducive to more efficient boundary recovery of objects. The output features were added with a full convolution layer to reduce them into feature maps that contained class_nums classifications and had the same size as input images.

#### 3.2.3. Feature Engineering

**Figure 5:**
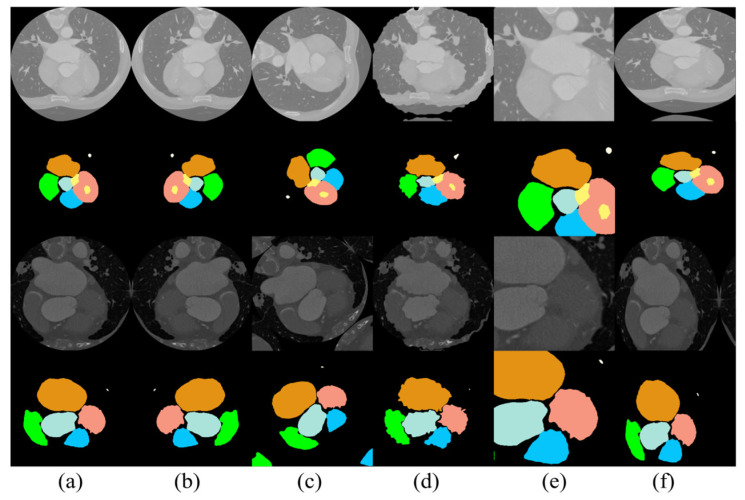
Results of data enhancement: (**a**) original image, (**b**) image after horizontal flip, (**c**) image after arbitrary rotation, (**d**) image after distortion, (**e**) image after random cropping, and (**f**) image after elastic distortion transformation.

The medical data available that can be employed for scientific research are quite limited, since the disclosure of medical datasets involves the personal privacy of patients, and the labeling of medical data consumes more labor and material resources compared with that of conventional data. To maximize the utilization of limited data, existing datasets were subjected to image enhancement in this study. The creation of new training samples based on existing data reduced the probability of overfitting caused by a lack of data and improved the final experimental results to some extent. In this model, the images were mainly subjected to horizontal rotation, vertical rotation, arbitrary rotation, distortion, rotational translation, random cropping, and elastic distortion transformation. The results of the data enhancement are presented in Figure 5.

## 4. Results and Discussions

### 4.1. Implementation Details

#### 4.1.1. Datasets

**Figure 6:**
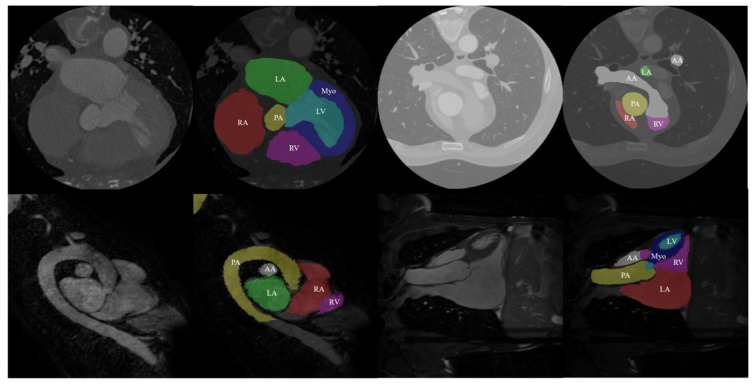
Data and true labels in the dataset.

The data used in this study came from the MM-WHS Challenge 2017 open-source dataset. This dataset includes 20 labeled cardiac CT image training sets, 20 labeled cardiac MRI image training sets, 40 unlabeled CT images, and 40 unlabeled MRI images. All data in this dataset are three-dimensional (3D) medical images with real value labels for the heart. Specifically, these labels include seven cardiac substructures: ascending aorta (AA), pulmonary artery (PA), left atrium (LA), left ventricle (LV), left ventricular myocardium (Myo), right atrium (RA), and right ventricle (RV) (as shown in Figure 6). The first row of Figure 6 displays the CT images and their corresponding real labels, while the second row displays the MRI images and their corresponding real labels.

Processing this dataset poses multiple challenges. Firstly, the complexity and diversity of 3D medical image data increase the difficulty of analysis and processing. Different imaging modalities (CT and MRI) have varying contrasts and resolutions, requiring the model to adapt to these variations. Additionally, the boundaries between cardiac substructures are often blurred, with complex shapes and overlapping structures further complicating the segmentation task.

During data processing, we first sliced the 3D data into 20 consecutive sets, each containing approximately 4000 cardiac CT image slices and 2500 cardiac MRI image slices. Considering that small differences between consecutive CT slices could lead to overfitting of the experimental results, we performed image scrambling and redundant data removal. These steps ensured data diversity, reduced redundant information, and enhanced the model’s generalization ability, thereby avoiding overfitting.

Dataset expansion also presented a significant challenge. To ensure the model had sufficient training data, we employed the method described in Section 3.2.3, expanding the number of images in the CT and MRI datasets to 20,000 and 9000, respectively. During the expansion process, we utilized various data augmentation techniques, such as rotation, translation, scaling, and flipping, to increase the diversity and richness of the data. These techniques helped us build a more robust dataset, enabling the model to learn and generalize better.

#### 4.1.2. Training

Among the resulting 20 sets of heart data, the first 15 sets were selected as training data, and the remaining 5 sets were used as test data. Only the training data were subjected to enhancement. Ten-fold cross-validation was employed for the training validation. The initial learning rate was set to 1 × 10^−4^. In all networks, the Adam optimizer was used for training at a batch size of four. The cross-entropy loss function shown in Formula (7) was adopted as the loss function, where *x* is the result predicted by the model, and *y* is the corresponding real label.

(7) $$C=-\frac{1}{n}\sum_{x}\left[y\ln a+(1-y)\ln(1-a)\right]$$

Detailed information about the server used in this study is as follows: GeForce Titan Xp (12G video memory), GeForce Titan V (12G video memory), Intel Xeon (R) CPU E5-1620, and Ubuntu16 system. Python 3.7.5 and Pytorch 1.7.1 framework were utilized. A round of training took 40 min on average.

#### 4.1.3. Evaluation Indexes

The Mean Intersection over Union (MIoU) and Dice similarity coefficient were employed as evaluation metrics to assess the performance of our segmentation model. These metrics quantify the overlap between the predicted results and the ground truth, providing insight into the model’s accuracy.

MIoU measures the ratio of the intersection to the union of the predicted segmentation (*A*) and the ground truth (*B*) for each class. It is computed as follows:

(8) $$MIoU=\frac{1}{n}\sum_{1}^{n}\frac{\left|A\cap B\right|}{\left|A\cup B\right|}$$

where *A* represents the predicted segmentation for class. *B* represents the ground truth segmentation for class. *n* is the number of classes.

MIoU values range from 0 to 1, with higher values indicating better performance. A higher MIoU signifies a greater overlap between the predicted segmentation and the ground truth, reflecting more accurate predictions by the model.

The Dice similarity coefficient also evaluates the overlap between the predicted segmentation and the ground truth but gives more weight to the size of the overlapping regions. It is defined as:

(9) $$Dice=\frac{2\left|A\cap \left.B\right|\right.}{\left|A\right|+\left|B\right|}$$

|*A* ∩ *B*| denotes the area of overlap between the predicted segmentation and the ground truth, |*A*| is the area of the predicted segmentation, and |*B*| is the area of the ground truth segmentation.

The Dice coefficient ranges from 0 to 1, with higher values indicating better performance. A higher Dice coefficient means a larger proportion of the predicted segmentation matches the ground truth, demonstrating more precise segmentation results.

### 4.2. Comparisons with Other Advanced Methods

**Figure 7:**
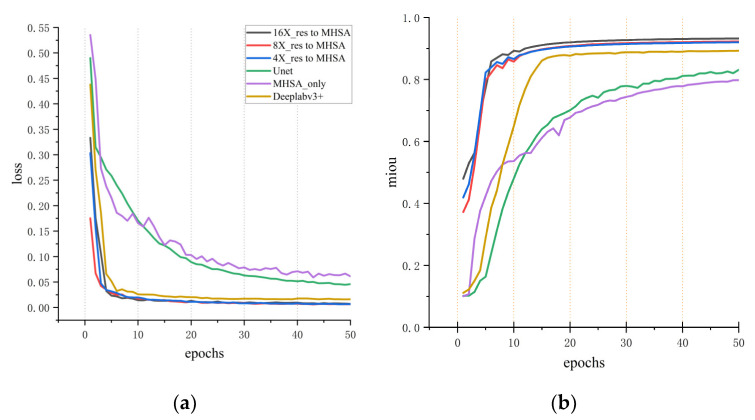
Comparisons with other methods for cardiac CT segmentation results. (**a**) The variation of loss with respect to epochs for different models. (**b**) The variation of mIoU (mean Intersection over Union) with respect to epochs.

In the whole heart segmentation task described above, several experiments were conducted to compare the method proposed in this study with other advanced methods. The segmentation results on different indexes for cardiac CT and MRI images are summarized in Table 1, Table 2, Table 3 and Table 4. Initially, the method proposed in this study was compared with advanced methods, including Segmenter, U-net, and DeepLab V3+ network, for MIoU performance on cardiac CT datasets (Table 1). For the sake of fairness, datasets with augmented data were used for comparison, and the training strategy and experimental equipment were the same. To accelerate the training of networks, the pre-training DeepLab V3+ model for CT data was imported into the model proposed in this study. As shown in Table 1, the MIoU of the proposed three networks at different scales topped out at 93.7%. It can also be seen from Figure 7a,b that the MSMHSA-DeepLab V3+ network model proposed in this study outperformed the network models, which had not been improved in terms of either the convergence rate or the segmentation results.

The comparison of Dice segmentation performance between advanced segmentation models and the method proposed in this study is shown in Table 2, in which the first three are the top three segmentation indicators in the MM-WHS Challenge 2017 [26]. Table 2 shows there was a difference of 3.2% in the RA result between our model and Swin-Unet, but the model proposed in this study saw a great improvement in the average Dice value, peaking at 0.9374.

Table 3 displays the segmentation results of cardiac MRI images by the network model proposed in this study. In contrast with cardiac CT images, cardiac MRI images are of higher irregularity, and thus, their segmentation is more complex and difficult. Moreover, cardiac MRI images are not as intuitive or convenient to process as cardiac CT images. Currently, the segmentation of cardiac MRI images mainly involves two to three substructures of the heart, and whole heart segmentation is rare. The second network in Table 3 is the U-net modified with Masc for whole heart segmentation of MRI images. The results showed that the model proposed in this study achieved excellent segmentation results for cardiac MRI images compared with other methods despite a distinct gap in the segmentation results for CT images between the U-net modified with Masc and the model proposed in this study.

**Table 1:** CT image segmentation results in Miou metrics. (Bold values indicate the best performance).

IoU	Myo	LA	LV	RA	RV	PA	AA	MIoU
Segmenter	0.440	0.500	0.558	0.338	0.480	0.672	0.716	0.529
U-net	0.774	0.878	0.864	0.792	0.804	0.927	0.804	0.835
DeepLabV3+	0.858	0.921	0.914	0.885	0.885	0.937	0.858	0.894
ConvFormer [27]	0.857	0.885	0.914	0.835	0.836	0.740	**0.957**	0.861
16×_res to MHSA	0.868	**0.980**	0.898	**0.982**	0.902	**0.984**	0.943	**0.937**
8×_res to MHSA	**0.873**	0.969	0.901	**0.982**	**0.905**	0.968	0.906	0.929
4×_res to MHSA	0.872	0.975	**0.915**	0.969	0.887	0.944	0.915	0.925

**Table 2:** CT image segmentation results in Dice metrics. (Bold values indicate the best performance).

Dice	Myo	LA	LV	RA	RV	PA	AA	Avg
GUT	0.881	0.929	0.918	0.888	0.909	0.840	0.933	0.899
KTH [28]	0.856	0.930	0.923	**0.971**	0.857	0.835	0.894	0.881
CUHK1 [29]	0.851	0.916	0.904	0.836	0.883	0.784	0.907	0.869
3D U-net	0.791	0.853	0.813	0.909	0.816	0.763	0.717	0.8089
Two stage U-net	0.729	0.904	0.799	0.786	0.793	0.648	0.873	0.7903
CFUN	0.822	0.832	0.879	0.902	0.844	0.821	0.940	0.8590
SEG-CNN	0.872	0.91	0.924	0.879	0.865	0.837	0.913	0.8896
Swin-Unet	0.856	-	**0.958**	-	0.886	-	-	0.9000
MAUNet [30]	0.893	0.910	0.925	0.928	0.886	0.866	0.925	0.9063
ConvFormer	**0.926**	0.943	0.955	0.914	0.910	0.844	**0.980**	0.9250
16×_res to MHSA	0.899	**0.961**	0.917	0.962	0.919	**0.963**	0.941	**0.9374**
8×_res to MHSA	0.901	0.955	0.918	0.962	**0.921**	0.954	0.922	0.9333
4×_res to MHSA	0.900	0.958	0.926	0.955	0.912	0.943	0.927	0.9316

**Table 3:** MRI image segmentation results in Miou metrics. (Bold values indicate the best performance).

**IoU**	**Myo**	**LA**	**LV**	**RA**	**RV**	**PA**	**AA**	**MIoU**
U-net	0.5572	0.4087	0.6958	0.5282	0.6085	0.3354	0.1170	0.4644
MascParallelUnet	0.5835	0.6155	0.7271	0.5625	0.5361	0.6849	0.6556	0.6207
DeepLabV3+	0.7676	0.8129	0.8817	0.8403	0.8628	0.7989	0.7302	0.8135
ConvFormer	**0.857**	0.885	**0.914**	0.835	0.836	0.740	**0.957**	0.861
16×_res to MHSA	0.828	0.922	0.803	**0.934**	**0.879**	0.926	0.891	0.883
8×_res to MHSA	0.827	0.914	0.815	0.911	0.850	0.905	0.852	0.868
4×_res to MHSA	0.835	**0.930**	0.818	0.921	0.870	**0.928**	0.893	**0.885**

Table 4 presents the comparative results of cardiac MRI image segmentation tasks using the Dice metric in comparison to alternative methods. Since most whole heart segmentation methods primarily rely on CT images for training, the availability of MRI segmentation results for comparison is limited. Therefore, the comparative experiment includes nnUnet, nnFormer, MMGL, and MISSFormer networks for partial cardiac tissue segmentation in the evaluation of the Dice metric for whole heart segmentation. The experimental results show close performances across the nnUnet, nnFormer, MMGL, and MISSFormer models. Notably, MMGL excels in accurately segmenting the left ventricle (LV) among all the networks. Significantly, the integration of self-attention mechanisms at various scales in our model demonstrates superior performance compared to other models, as evidenced by the average Dice score of 0.938 achieved by our 4×_res to MHSA model. Moreover, employing larger and smaller scale self-attention mechanisms yields comparable average Dice scores, as shown in Table 4. The result also shows that despite the advanced results achieved by transformer models in medical image segmentation, our model proves to be simpler and more accurate for whole heart segmentation.

**Table 4:** MRI image segmentation results in Dice metrics. (Bold values indicate the best performance).

Dice	MYO	LA	LV	RA	RV	PA	AA	Avg
nnUnet [31]	0.892	-	0.953	-	0.902	-	-	0.916
nnFormer [32]	0.896	-	0.957	**-**	0.909	-	-	0.921
MMGL [33]	0.900	-	**0.961**	-	0.909	-	-	0.923
MISSFormer [34]	0.880	-	0.949	-	0.896	-	-	0.909
Unet	0.716	0.580	0.821	0.691	0.757	0.5023	0.209	0.611
MascParallelUnet	0.737	0.762	0.842	0.720	0.698	0.813	0.792	0.766
Deeplab V3+	0.869	0.897	0.937	0.913	0.926	0.888	0.844	0.896
MAUNet	0.893	0.910	0.925	0.928	0.886	0.866	0.925	0.9063
ConvFormer	**0.926**	0.943	0.955	0.914	0.910	0.844	**0.980**	0.925
16×_res to MHSA	0.906	0.959	0.891	**0.966**	**0.936**	0.962	0.942	0.937
8×_res to MHSA	0.905	0.955	0.898	0.953	0.919	0.950	0.920	0.929
4×_res to MHSA	0.910	**0.964**	0.900	0.959	0.930	**0.963**	0.943	**0.938**

### 4.3. Comparison between the Models at Different Scales

**Figure 8:**
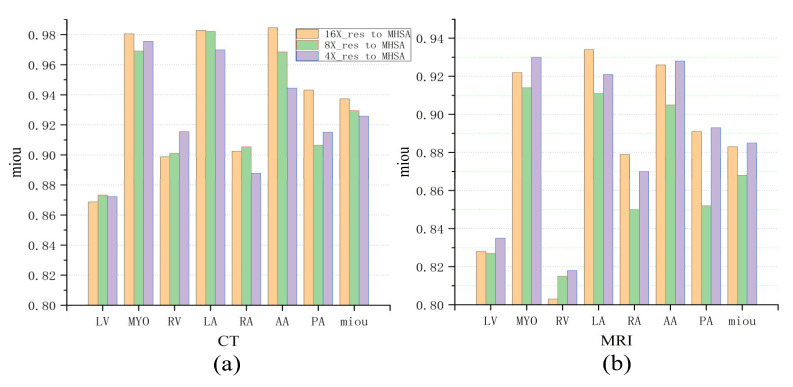
Comparison of three networks in cardiac CT and MRI image segmentation. (**a**) The mIoU (mean Intersection over Union) for CT images. (**b**) The mIoU for MRI images.

Figure 8a intuitively demonstrates the impact of MHSAs at three different scales on the final experimental results. In fact, this multi-scale comparison can be understood as the result analysis of multi-scale ablation experiments. As shown in the figure, for the larger left ventricle (LV), better segmentation results were obtained using MHSA at a larger scale (4×_res to MHSA) in both CT images and MRI images. However, for the smaller ascending aorta (AA) and pulmonary artery (PA), better results were achieved using MHSA at a smaller scale (16×_res to MHSA). The above results did not apply to the left ventricular myocardium (Myo) with its more complex morphology, and the differences among MHSA models at different scales were small. For the moderately sized right atrium (RA) and right ventricle (RV), better results were obtained using MHSA at a medium scale (8×_res to MHSA) and at a smaller scale (16×_res to MHSA), while for the left atrium (LA), better results were achieved using MHSA at a smaller scale (16×_res to MHSA).

In terms of MRI images, better segmentation results were obtained using MHSA at a larger scale [Figure 8b], which is contrary to the segmentation results for CT images. This is because the left atrium (LA), left ventricle (LV), and left ventricular myocardium (Myo) in the cardiac MRI dataset are much larger than the right atrium (RA) and right ventricle (RV) in size. Therefore, better segmentation results for the LA, LV, and Myo were achieved using the network at a smaller scale (16×_res to MHSA), and better segmentation of the RA and RV were acquired using the network at a larger scale (4×_res to MHSA) during segmentation. In Table 3, the 4×_res to MHSA network performed best for the segmentation of the larger LV, whereas the 16×_res to MHSA network performed best for the segmentation of the relatively smaller RA. For the segmentation of the smaller PA and AA, the difference was negligible between the two. The prediction results of the 4×_res to MHSA network were not as good as those of the other two networks.

To validate the effectiveness of MHSA at different resolutions, we designed and implemented statistical analyses. We conducted paired t-tests and calculated confidence intervals for the transitions from 16×_res to MHSA, 8×_res to MHSA, and 4×_res to MHSA.

In the paired *t*-tests, the *p*-value for the transition from 16×_res to MHSA and 8×_res to MHSA was 0.03, and the *p*-value for the transition from 8×_res to MHSA and 4×_res to MHSA was 0.009. Both *p*-values are less than 0.05, indicating that the results are statistically significant.

### 4.4. Segmentation Results

**Figure 9:**
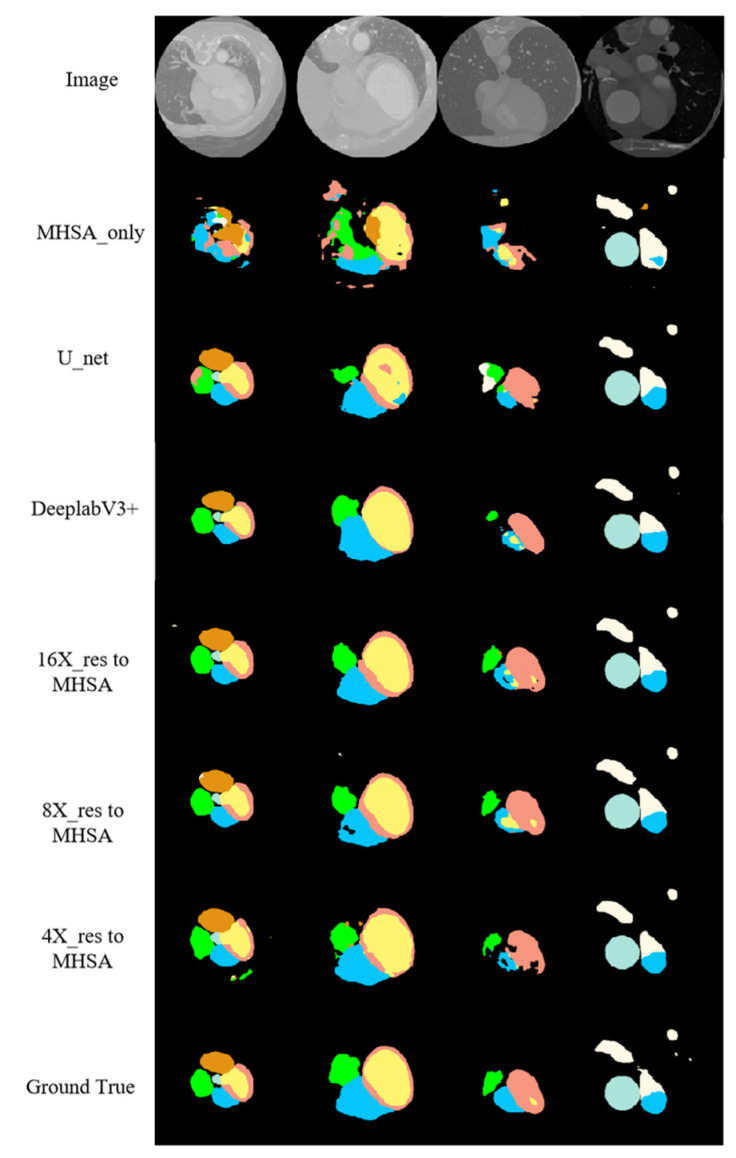
CT image segmentation results.

Figure 9 shows the segmentation results of the proposed networks on the cardiac CT image test set along with the segmentation results of the MHSA mechanism only, U-net, and DeepLab V3+. For CT images, the application of the self-attention mechanism in the 16-fold down-sampling results achieved better similarity to the real labels, but for substructures with a smaller size, the results were still lacking. However, the proposed networks had quite a good advantage over DeepLab V3+ and other networks. Moreover, the proposed networks were more accurate in predicting the boundary of the AA (the white region in Figure 9) and had visually better predicted results for the LV (the pink region in Figure 9) than those by other networks.

**Figure 10:**
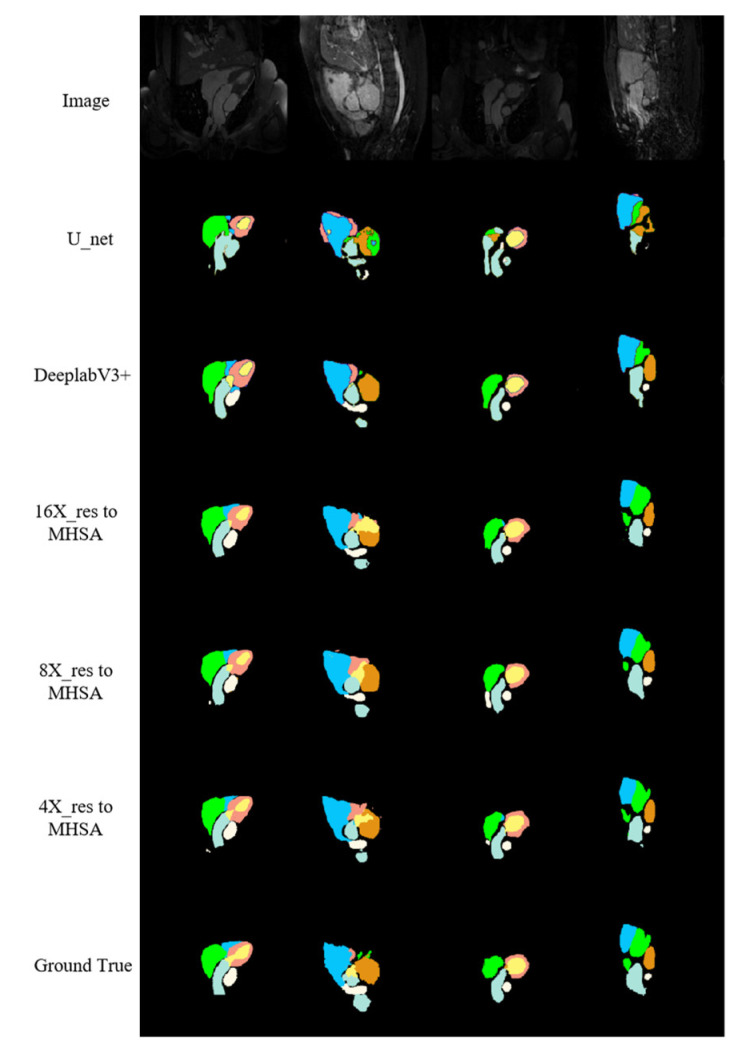
MRI image segmentation results.

According to the segmentation results of networks on the cardiac MRI image test set along with the other relevant networks shown in Figure 10, cardiac MRI images were darker than cardiac CT images, and the position of the heart was not obvious enough, giving a more complex and variable shape for the heart. The proposed networks performed better in predicting the RV (the blue region in Figure 10) than other methods. Additionally, the contour of the LA (the green region in Figure 10) had closer similarity to the real label, especially when at large scale (the green region in the fourth column). The proposed networks were more accurate than other networks in recognizing the details of the boundaries, the number of structures finally predicted, and the positional relationships between structures.

## 5. Conclusions

In this study, we propose an efficient whole heart segmentation network based on multi-scale, multi-head self-attention (MSMHSA) mechanisms. This network enables the acquisition of larger receptive fields and the extraction of richer semantic information. We seamlessly integrate the MSMHSA mechanisms into DeepLab V3+, leveraging its capability to extract rich context information, and complement it with jumping connections in the decoder to enhance its suitability for medical image segmentation. This strategic design significantly improves boundary recovery in images. We conducted extensive experiments on publicly available cardiac CT and MRI datasets to validate the effectiveness of our proposed MSMHSA-DeepLabV3+ model. The results demonstrate significant advancements in multiple leading-performance indexes for dual-modality cardiac medical images simultaneously, confirming the effectiveness and generality of our proposed method.

To further expand the model’s applicability, we plan to apply it to other medical datasets, such as the Synapse dataset for abdominal multi-organ segmentation. Additionally, we aim to increase the complexity of the network in the future. Currently, the model only utilizes simple jumping connections, but we may introduce more complex jumping connection designs. For instance, we may refer to the nested and dense jumping connection designs in U-Net++ [35] and U-Net3+ [36] or adopt the MultiRes module in MultiResUNet [37]. Through these improvements, we expect our model to demonstrate stronger generality and superiority in a wider range of medical image segmentation tasks.

## Data Availability

The data used in this study are openly available at https://zmiclab.github.io/zxh/0/mmwhs/ (accessed on 30 May 2024).
